# Coumarin derivatives ameliorate the intestinal inflammation and pathogenic gut microbiome changes in the model of infectious colitis through antibacterial activity

**DOI:** 10.3389/fcimb.2024.1362773

**Published:** 2024-07-15

**Authors:** Hui-su Jung, Yei Ju Park, Bon-Hee Gu, Goeun Han, Woonhak Ji, Su mi Hwang, Myunghoo Kim

**Affiliations:** ^1^ Laboratory of Animal Immunology, Department of Animal Science, College of Natural Resource & Life Science, Pusan National University, Miryang, Republic of Korea; ^2^ R & D Center, EyeGene, Goyang, Republic of Korea; ^3^ Life and Industry Convergence Research Institute, Pusan National University, Miryang, Republic of Korea; ^4^ Future Earth Research Institute, PNU JYS Science Academy, Pusan National University, Busan, Republic of Korea; ^5^ Department of Biomedical Laboratory Science, College of Health and Medical Science, Sangji University, Wonju, Republic of Korea

**Keywords:** coumarin derivative, antibacterial activity, anti-inflammation, gut immunity, gut microbiome

## Abstract

Coumarin, a phenolic compound, is a secondary metabolite produced by plants such as Tanga and Lime. Coumarin derivatives were prepared via Pechmann condensation. In this study, we performed *in vitro* and *in vivo* experiments to determine the antimicrobial and gut immune-regulatory functions of coumarin derivatives. For the *in vitro* antimicrobial activity assay, coumarin derivatives C1 and C2 were selected based on their pathogen-killing activity against various pathogenic microbes. We further demonstrated that the selected coumarin derivatives disrupted bacterial cell membranes. Next, we examined the regulatory function of the coumarin derivatives in gut inflammation using an infectious colitis model. In an *in vivo* infectious colitis model, administration of selected C1 coumarin derivatives reduced pathogen loads, the number of inflammatory immune cells (Th1 cells and Th17 cells), and inflammatory cytokine levels (IL-6 and IL-1b) in the intestinal tissue after pathogen infection. In addition, we found that the administration of C1 coumarin derivatives minimized abnormal gut microbiome shift-driven pathogen infection. Potential pathogenic gut microbes, such as *Enterobacteriaceae* and *Staphylococcaceae*, were increased by pathogen infection. However, this pathogenic microbial expansion was minimized and beneficial bacteria, such as *Ligilactobacillus* and *Limosilactobacillus*, increased with C1 coumarin derivative treatment. Functional gene enrichment assessment revealed that the relative abundance of genes associated with lipid and nucleotide metabolism was reduced by pathogen infection; however, this phenomenon was not observed in C1 coumarin derivative-treated animals. Collectively, our data suggest that C1 coumarin derivative is effective antibacterial agents that minimize pathogen-induced gut inflammation and abnormal gut microbiome modulation through their antibacterial activity.

## 1 Introduction

Mucosal tissue, including the gastrointestinal (GI) tract, respiratory tract, and reproductive organs, is the largest component of the immune system in the body (Mowat, 2003). Up to 70–80% of immune cells exist in the mucosal tissue, suggesting the importance of mucosal immunity (Pabst et al., 2008). Because mucosal tissue has a large surface area and is exposed to environmental materials such as pathogens, it is difficult to maintain immune homeostasis in the tissue (Bischoff, 2009). The GI tract is a part of the mucosal tissue and is composed of a large number of microbes and dietary antigens. The GI tract is the major entry site for various pathogens such as *Salmonella* and *Escherichia coli* (Muniz et al., 2012). Colonization of invasive pathogenic microbes damages gut tissue and causes dysbiosis of the gut microbiota, resulting in impaired intestinal tissue homeostasis (Coburn et al., 2007). Pathogen infection activates the innate and adaptive immune systems (Cao et al., 2021), leading to intestinal inflammation (Klag et al., 2013). In addition, some pathogens can invade the intestine and cause systemic inflammation (Hapfelmeier and Hardt, 2005). Therefore, to maintain gut and systemic health, it is essential that the intestinal immune system responds appropriately to pathogens.

Microbial infections are becoming a pressing issue for global health and the economy (Abraham et al., 2016). In recent years, the treatment of microbial infections has become a significant challenge with conventional antibiotic therapy (Suresh et al., 2017). Standard therapies, including immunosuppressive and biological treatments, are widely used, but these therapeutic agents have limitations. In particular, the effectiveness of existing antibacterial drugs has decreased or even become ineffective owing to microbial resistance (Baym et al., 2016). Recently, various studies have been conducted to overcome these problems, and innovative treatment methods using non-antibiotic drugs with potential antibacterial properties have attracted attention (Vandevelde et al., 2016). Therefore, efforts have been made to develop medicinal compounds derived from natural sources, such as fungi, plants, and animals (Rasheed et al., 2022). Several studies have suggested that these compounds can be used to treat intestinal inflammatory diseases (Maurya and Mishra, 2022).

Coumarin, a phenolic compound, is a secondary metabolite produced by plants such as Tanga (Hoult and Payá, 1996), cinnamon (Thada et al., 2013), and many types of medicinal plants including all parts of plants-fruits, roots, stems, and leaves (Lončar et al., 2020). The benzopyrone structure of coumarin enables its derivatives to readily interact with diverse enzymes and receptors in organisms (Ibrar et al., 2018). Coumarin derivatives have potential bioactivities because various substituents can be attached to various positions on the benzene ring (Flores-Morales et al., 2023). Coumarin and its derivatives exhibit a wide range of biological activities, including antiviral (Mishra et al., 2020), antifungal (Song et al., 2017), anti-inflammatory (Luchini et al., 2008), antitumor (Liu et al., 2014), antioxidant (Kadhum et al., 2011), and antibacterial properties (Flašík et al., 2009). Intestinal inflammation is regulated by host immunity and gut microbiota (Zheng et al., 2020). The crosstalk between the gut microbiome and immunity plays a critical role in maintaining gut tissue homeostasis (Campbell et al., 2023). Although the diverse functions of coumarins are known, there are only a few *in vivo* studies on the effects of coumarin derivatives on intestinal inflammation (Witaicenis et al., 2014). In this study, we examined the effects of coumarin derivatives on the regulation of intestinal inflammation and gut microbiome. We hypothesized that coumarin derivatives regulate intestinal inflammation and modulate the gut microbiome through antibacterial activity. In this study, the disc diffusion method was used to select effective coumarin derivatives from the various types of coumarin derivatives. Some coumarin derivatives exhibited pathogen-killing activity by disrupting bacterial cell membranes. Using the *Citrobacter rodentium (C. rodentium)* infectious colitis model, we further demonstrated that the selected coumarin derivatives (C1 and C2) have anti-inflammatory functions and can minimize pathogenic gut microbiome shifts through antibacterial activity. Our data suggest that coumarin derivatives may be effective therapeutic agents for suppressing intestinal inflammation and changes in the gut microbiome caused by pathogens via their antibacterial activity.

## 2 Materials and methods

### 2.1 Preparation of coumarin derivates

Representative methods for synthesizing coumarin derivatives include the Perkin reaction (He et al., 2014), Knoevenagel condensation reaction (Abraham et al., 2016), and Pechmann condensation reaction (Jadhav et al., 2019). In this study, natural coumarin was isolated from lime (*Citrus aurantifolia*) peel, separated, and purified. Afterwards, the material was identified through structural analysis, and various coumarin derivatives were synthesized via the Pechmann condensation reaction. A mixture containing ethyl acetoacetate, trifluoromethyl acetoacetate, and phenol derivatives was prepared, and sulfuric acid was added while maintaining the temperature at 10 °C. The mixture was stirred at room temperature for 18–26 hours, and then the temperature was maintained below 10 °C. The resulting precipitate was filtered, washed four times, and dried. Unrefined solids were purified by column chromatography using dichloromethane: n-hexane (5:1, v/v) as the eluent, and the structural analysis and yield were confirmed.

### 2.2 Microbial strains

To investigate the antimicrobial activity of coumarin derivatives through *in vitro* screening, 11 types of microbes were cultured according to the culture conditions for each strain*. Candida albicans* (11282, KCCM, Seoul, Republic of Korea) was cultured at 25 °C in YPD (yeast 1% + dextrose 2% + bacto Peptone 2%) media. *Bacillus cereus* (11204, KCCM) and *Micrococcus luteus* (1056, IAM, Tokyo, Japan) were cultured at 30 °C in ENB (yeast extract 0.25%+brain heart infusion broth 1.25% + nutrient broth 0.55%) media. *Enterococcus faecium* (12118, KCCM), *Listeria monocytogenes* (40307, KCCM), and *Streptococcus mutans* (3056, KCTC, Seoul, Republic of Korea) were cultured at 37 °C in brain heart infusion broth. *Salmonella enteritids* (12021, KCCM) and *Shigella boydii* (41649, KCCM) were cultured at 37 °C in nutrient broth media. *Escherichia coli* (11835, KCCM), *Staphylococcus aureus subsp.* (40050, KCCM), and *Citrobacter rodentium* (DBS 100, ATCC, Virginia, USA) were cultured at 37 °C in Luria-Bertani (LB) broth media.

### 2.3 Disc diffusion assay

The antimicrobial activity of different coumarin derivatives against several strains was tested using a previously reported disc diffusion method (Wang et al., 2020). Briefly, 30 ml LB medium (BD Biosciences, Heidelberg, Germany) was added to each petri dish. After solidification, 200 µl of each microbial suspension with a concentration of 6.4×10^6^ CFU/ml was uniformly coated on petri plates. Subsequently, a well of 8 mm size was made on the plate, and the concentration of each coumarin derivative was made up to 20 mg/ml and loaded into the well. After incubation at 37 °C for 18 hours, the diameter of the inhibition zone (DIZ) was measured to determine the antimicrobial activity of coumarin derivatives, and dimethyl sulfoxide (DMSO) was used as a negative control. The results were as follows according to the length of the DIZ: -, no activity; +, inhibition zone>5–10 mm; ++, inhibition zone>11–15 mm; +++, inhibition zone>16–20 mm; and ++++, inhibition zone>20 mm.

### 2.4 Confocal laser scanning microscopy analysis

Damage to the *C. rodentium* membrane was determined as described previously (Harhala et al., 2021), with some modifications. The bacterial pellets of *C. rodentium* were prepared at a concentration of 5×10^8^ CFU, based on 2.2. The test strains were treated with coumarin derivatives dissolved in DMSO at 10 mg/ml, except for the control group. DMSO was used as a negative control. After a 6 hours incubation period at 37 °C, the samples were washed twice with phosphate-buffered saline (PBS). The cell pellet was resuspended in 5 µM dye buffer using SYTOX™ Green Nucleic Acid Stain kit (Thermo Fisher Scientific, Waltham, MA, USA) and incubated at 37 °C in the dark for 15 minutes. Finally, 20 µl of the cell suspension was placed on a glass slide, and fluorescent images were acquired using a fluorescence microscope.

### 2.5 SEM analysis of bacteria

The morphological properties of the *C. rodentium* were assessed via scanning electron microscope (SEM). The coumarin derivatives-treated *C. rodentium* were prepared identically to 2.4. After washing the sample with PBS, the bacteria were fixed in 2.5% glutaraldehyde, post-fixed using 1% OsO_4_ for 1 hour, dehydrated in 50%, 70%, 95%, and 100% ethanol solutions, and then dried. The samples were coated with a sputter coater MC 1000 (Hitachi, Tokyo, Japan) and the examinations were carried out on S-4800 SEM (Hitachi, Tokyo, Japan).

### 2.6 *C. rodentium* infection and measurement of bacterial load in colon and cecum

Female C57BL/6N mice (eight-week-old) were orally inoculated with *C. rodentium* (strain DBS100) to induce colitis. Briefly, bacteria were grown overnight in LB and resuspended in PBS before infecting the mice (100 µl/mouse; 1×10^9^ CFU of *C. rodentium*). To evaluate the colonization of *C. rodentium*, fecal pellets from mice were collected daily at intervals after infection and cecal pellets were collected after sacrificing the mice. They were then weighed, homogenized, serially diluted to equal concentrations, and plated on MacConkey agar plates. After incubation at 37°C for 18 hours, the bacterial colonies were counted.

### 2.7 Coumarin derivatives treatment

Following the initial acclimation period, the mice were randomly divided into four groups: (1) non-infected group (Control group, n=5) (2) *C. rodentium*-infected group (Citro group, n=5), (3) C1 treated and *C. rodentium*-infected group (Citro+C1 group, n=5), and (4) C2 treated and *C. rodentium*-infected group (Citro+C2 group, n=5). For the C1 group, all mice were administered C1 coumarin derivates dissolved in aqueous NaOH solution at 20 mg/ml (2 mg/mouse, 100 µl/mouse) via oral gavage once a day for 7 days. For the C2 group, all mice were administered C2 coumarin derivates dissolved in aqueous NaOH solution at 20 mg/ml (2 mg/mouse, 100µl/mouse) via oral gavage once a day for 7 days. After two days of intervention, all mice except the control group were infected with *C. rodentium* (100 µl/mouse; 1×10^9^ CFU).

To study the efficacy of coumarin derivatives in gut microbiome modulation, another experiment was conducted using 8-week-old female C57BL/6N mice. After the initial acclimation period, the mice were randomly divided into three groups, with five mice per group. The treatment groups were as follows: (1) non-infected (Control group, n=5), (2) *C. rodentium*-infected (Citro group, n=5), and (3) C1 treated and *C. rodentium*-infected groups (Citro+C1 group, n=5). For the C1 group, all mice were administered C1 coumarin derivates dissolved in aqueous NaOH solution at 20 mg/ml (2 mg/mouse, 100 µl/mouse) via oral gavage once a day for 7 days. After two days of intervention, groups (2) and (3) were infected with *C. rodentium* (100 µl/mouse; 1×10^9^ CFU). Body weights (BW) were measured on days -3, 0, 3, 6, 9, and 12 after *C. rodentium* infection, and all mice were sacrificed 12 days post infection.

### 2.8 Quantitative detection of colonic cytokine expression

Quantitative real-time PCR (qRT-PCR) was performed to quantify the expression analysis of IL-6, IL-1b, and IL-10. Total RNAs were isolated from mouse colon tissue. It homogenized at 6 m/s for 30 s and the homogenate was centrifuged. The supernatant was mixed with chloroform (Sigma Aldrich, Chem, USA) and the mixture was incubated for 3 minutes at room temperature. The supernatant was obtained through centrifugation and mixed with isopropanol (Biosesang, Seoul, Republic of Korea). The sample was centrifuged to remove the supernatant and 75% DEPC-EtOH made from DEPC-treated water and EtOH (Bioseang, Seoul, Republic of Korea) was added. Isolated RNA was used for cDNA synthesis using AccuPower^®^ RT PreMix (Bioneer, Daejeon, South Korea) following the manufacturer’s instructions. To confirm mRNA expression levels, qRT-PCR was performed using a QuantStudio 1 Real-Time PCR system (Applied Biosystems, Waltham, CA USA) with the following reaction conditions: 50 °C for 2 min, 95 °C for 15 min, 95 °C for 20 s, 60°C for 40 s, and 72 °C for 20 s (40 cycles), followed by melting curve analysis. GAPDH was used as the housekeeping gene, and relative quantification was calculated using △△Ct method. The primers used for gene amplification were as follows: GAPDH (forward primer 5′- ATCCTGCACCACCAACTGCT-3′ and reverse primer 5′- GGGCCATCCACAGTCTTCTG-3′), IL-6 (forward primer 5′- CCAGAGATACAAAGAAATGATGG-3′ and reverse primer 5′- ACTCCAGAAGACCAGAGGAAAT-3′), IL-10 (forward primer 5′- CATCATGTATGCTTCTATGCAG-3′ and reverse primer 5′- CCAGCTGGACAACATACTGCT-3′), and IL-1b (forward primer 5′- CCTGGGCTGTCCTGATGAGAG-3′ and reverse primer 5′- TCCACGGGAAAGACACAGGTA-3′).

### 2.9 Cell isolation of lamina propria and mesenteric lymph node

Colonic LP cells were isolated as previously described (Diehl et al., 2013). Briefly, the obtained intestinal tissue was placed in PBS, open, and the luminal contents were removed. Intestines were cut into 1 cm sections and then treated with 1 mM DL-dithiothreitol (DTT; Sigma-Aldrich, Irvine, UK), 30 mM ethylenediaminetetraacetic acid (EDTA; Thermo Fisher Scientific, Waltham, MA, USA), and 10 mM 4-[2-hydroxyethyl]-1-piperazineerhanesulfonic acid (HEPES; Thermo Fisher Scientific, Waltham, MA, USA), and incubated at 37°C for 10 minutes to remove mucus. The incubated tissue was treated with 30 mM EDTA and 10 mM HEPES in the same manner as in the previous process to remove epithelial cells. Tissue was then digested in 0.5 mg/ml collagenase (Sigma-Aldrich, Chem, Fort Lauderdale, FL, USA) and 150 µg/ml DNase I (Sigma-Aldrich, Chem, Fort Lauderdale, FL, USA) in RPMI supplemented with 10% FBS while shaking at 37°C for 1 h, followed by separation on a 40%/80% Percoll (Cytiva, Marlborough, MA, USA) gradient. LP cells were obtained by isolating cells between the two layers.

MLN cells were also used to confirm the changes in the distribution of immune cells. Briefly, MLN tissue was placed in PBS, ground, and passed through a strainer to remove debris. Among the cells from which debris was removed, ammonium chloride potassium was used to remove red blood cells. And MLN cells were acquired after washing with PBS.

### 2.10 Flow cytometry analysis

Isolated LP and MLN cells were measured using a FACS Canto II (BD Biosciences, Heidelberg, Germany) and analyzed using FlowJo software v10.7.1 (Tree Star Inc., Ashland, OR, USA). The LIVE/DEAD fixable aqua dead cell stain kit (Thermo Fisher Scientific, Waltham, MA, USA) was diluted and stained with an antibody to analyze only living cells. The antibodies used for staining targeted cells in this study are as follows: CX3CR1 (SA011F11, BioLegend, San Diego, CA, USA), CD11b (M1/70, BioLegend), MHC II (M5/114.15.2, BioLegend), Ly6C (1A8, BioLegend), CD3 (145-2C11, BioLegend), CD4 (RM4-5, BioLegend), CD8 (53-6.7, BioLegend), T-bet (4B10, Invitrogen, Eugene, OR, USA), RORgt (B2D, Invitrogen), and Foxp3 (FJK-16s, Invitrogen).

### 2.11 Gut microbiome analysis

The total microbial genomic DNA of each cecal content sample was extracted using the DNeasy PowerSoil Kit (Qiagen, Hilden, Germany) according to the manufacturer’s protocol, and the extracted DNA was quantified using Quant-IT PicoGreen (P7589, Invitrogen, Eugene, OR, USA). Sequencing libraries were prepared according to the Illumina 16S metagenomic sequencing library protocol to amplify the V3 and V4 regions. Briefly, 2 ng of DNA was amplified using 5X reaction buffer, 1 mM dNTP mix, 500 mM of each general F/R RCP primer, and Herculase II fusion DNA polymerase (Agilent Technologies, Santa Clara, USA). The general primer set utilizing the Illumina adapter overhang sequence used for PCR was as follows: V3-F: 5′-TCGTCGGCAGCGTCAGATGTGTATAAGAGACAGCCTACGGGNGGCWGCAG-3′, V4-R: 5′-GTCTCGTGGGCTCGGAGATGTGTATAAGAGACAGGACTACHVGGGTATCTAATCC-3′. The PCR products were purified using AMPure beads (Agencourt Bioscience, Beverly, MA, USA) and quantified by qPCR according to the manufacturer’s protocol (KAPA library quantification kits for Illumina Sequencing platforms). Paired-end (2×300 bp) sequencing was performed using the MiSeq platform (Macrogen, San Diego, CA, USA).

After sequencing, the raw data for each sample were aligned using an index sequence to determine the microbial characteristics. A paired-end FASTQ file was created for each sample, and sequencing errors were corrected by cutting the F/R primers and chimeric sequences using DADA2 (v.18.0). 300 bp and 250 bp of the forward and reverse sequences, respectively, were removed, and the amplicon sequence variant ASV was generated using the consensus method of DADA2. Additionally, subsampling and normalization were performed using the QIIME2 program for comparative analysis of microbial clusters. Each ASV sequence was assigned taxonomic information to the microorganism with the highest similarity, using reference databases. Alpha diversity analysis (Observed features, Chao1, Shannon, and Simpson), an indicator of the diversity and uniformity of microorganisms in cecal samples, were performed using QIIME with ASV abundance and taxonomic information. Principal coordinate analysis (PCoA) was used to visualize the relationships between samples and reveal beta diversity. LEfSe analysis was conducted to identify significant differences in the microbiome between the two groups. Functional gene assessment of the microbes was classified based on the Greengenes (v.13.5) and performed using PICRUSt 2 (Douglas et al., 2018), and metagenomic information was classified based on the Kyoto Gene and Genome Encyclopedia (KEGG) database. The threshold of the Kruskal–Wallis test was 0.05, and algebraic linear discriminant analysis score (LDA) over 2.0 were selected. Functional shifts inferred from microbiome- and genus-level taxonomic contributions were obtained using Python-based Functional Shifts’ Taxonomic Contributors (FishTaco) (Manor and Borenstein, 2017). FishTaco was used to infer the taxonomic and functional richness profiles from the PICRUSt analysis. Metagenomic- and taxonomic-based functional shifts were calculated using comparative functional analysis between samples, and the functional shifts were decomposed into genus-level contributions. The output was visualized using the FishTaco Plot package (https://borenstein-lab.github.io/fishtaco/visualization.html).

### 2.12 Statistical analysis

All experimental data are presented as mean ± standard deviation. Statistical differences between the two groups were determined using a two-tailed Student’s t-test. Datasets involving the three or four groups were assessed using one-way analysis of variance followed by Tukey’s multiple comparison test. Statistical analyses were performed using the GraphPad Prism 8 software (GraphPad, La Jolla, CA). Statistical significance was set at P<0.05.

## 3 Results

### 3.1 Synthesis of coumarin derivatives with various structures

The chemical compositions of the five coumarin derivatives synthesized for use in this study are listed in **Table 1**. The structures of all coumarin derivatives are based on C_9_H_6_O_2_ and represent different classes of compounds depending on their functional groups. 7-hydroxy-4-2*H*-chromen-2-one was prepared using the C_10_H_5_F_3_O_3_ formula with a molecular weight of 230.14 and having a CF_3_ group. In addition, 4-methylesculetin with the C_9_H_6_O_3_ formula and a molecular mass of 192.17 was synthesized to have the characteristic of having a CH_3_ group. Umbelliferone, dicumarol, and 4-hydroxycoumarin were synthesized using the chemical formulas C_9_H_12_O_6_, C_19_H_12_O_6,_ and C_9_H_6_O_3_, with molecular weights of 162.14, 336.3, and 162.14, respectively. The color of all coumarin derivatives, except 7-hydroxy-4-2*H*-chromen-2-one (yellow), was white. And the coumarin type of all coumarin derivatives, except dicumarol, which is biscoumarin, was confirmed to have a single coumarin structure.

**Table 1:** Chemical properties of synthesized coumarin derivatives.

Compound	Structure	Chemical formula	Name	Molecular weight	Color
**C1**	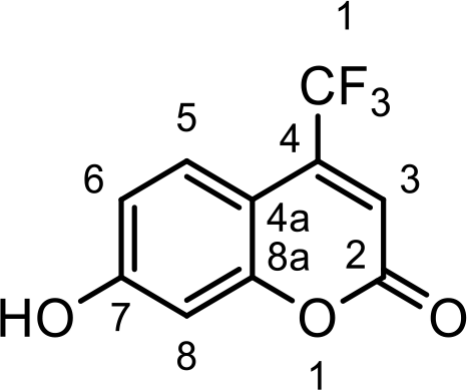	C_10_H_5_F_3_O_3_	7-hydroxy-4- (trifluoromethyl)- 2*H*-chromen-2-one	230.1401	yellow
**C2**	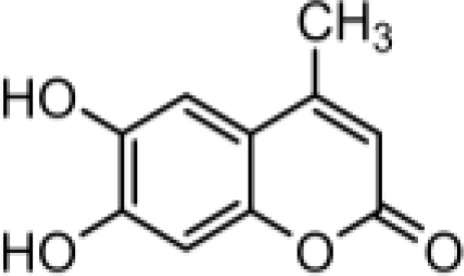	C_10_H_8_O_4_	4-methylesculetin	192.1681	white
**C3**	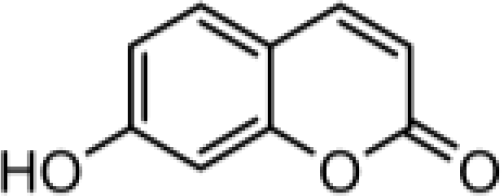	C_9_H_6_O_3_	Umbelliferone	162.1421	white
**C4**	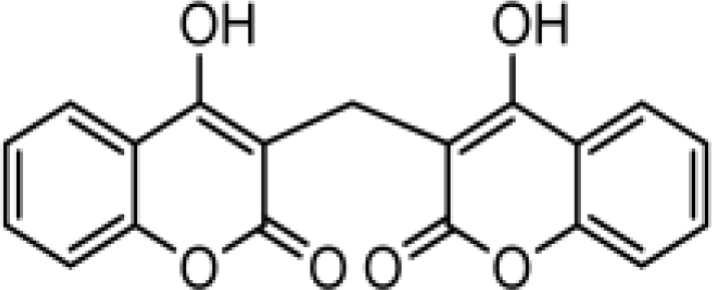	C_19_H_12_O_6_	Dicumarol	336.2949	white
**C5**	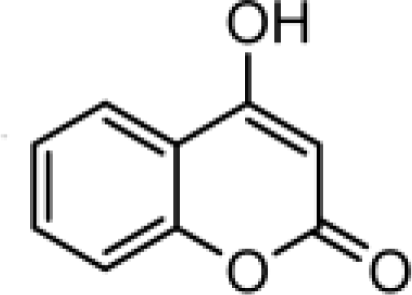	C_9_H_6_O_3_	4-hydroxycoumarin	162.1421	white

### 3.2 Identification of antimicrobial activities of coumarin derivatives

The antimicrobial efficacies of coumarin derivatives against 11 microbial strains were evaluated using the disc diffusion method by determining the surrounding zones of inhibition (**Table 2**). Antimicrobial activities were examined after adjusting the concentrations of the five types of samples to 20 mg/ml. The C1 coumarin derivatives produced an inhibition zone in all types of microbes; in particular, the inhibition zone in *Bacillus cereus* KEEM 11204 showed the highest activity with a size of 20 mm or more. The C2 coumarin derivative exhibited antimicrobial activity against eight types of microbes. The C3 derivative exhibited antimicrobial activity only against *C. albicans* KCCM 11282 and *B. cereus* KEEM 11204. The C4 coumarin derivative showed the highest activity against *B. cereus* KEEM 11204 and *S. aureus subsp. aureus* KCCM 40050. In addition, the C5 coumarin derivative showed good antibacterial activity against *B. cereus* KEEM 11204 and *L. monocytogenes* KEEM 40307. The growth inhibition zones of C1, C2, and C5 derivatives against *C. rodentium* DBS 100 showed good activity in the 11–15 mm range. In addition, it was confirmed that the solvent had no effect on the inhibitory activity, as no growth inhibition zone was formed around the well injected with DMSO as a negative control. Collectively, these results indicated that C1, C2, and C5 coumarin derivatives have effective functions.

**Table 2:** Antimicrobial activities of coumarin derivatives against 11 pathogens.

Test organisms	DMSO	C1	C2	C3	C4	C5
***C. albicans KCCM 11282***	**-**	**+**	**++**	**+**	**+**	**+**
***B. cereus KEEM 11204***	**-**	**++++**	**-**	**++++**	**++++**	**++**
***M. luteus IAM 1056***	**-**	**+++**	**++**	**-**	**+++**	**-**
***L. monocytogenes KEEM 40307***	**-**	**++**	**+**	**-**	**++**	**++**
***E. faecium KCCM 12118***	**-**	**++**	**++**	**-**	**++**	**+**
***S. mutans KCTC 3065***	**-**	**+++**	**-**	**-**	**+++**	**+**
***S. enteritidis KCCM 12021***	**-**	**++**	**++**	**-**	**-**	**-**
***S. boydii KCCM 41649***	**-**	**++**	**++**	**-**	**-**	**-**
***E. coli KCCM 11835***	**-**	**+**	**++**	**-**	**-**	**+**
***S. aureus subsp. aureus KCCM 40050***	**-**	**+++**	**-**	**-**	**++++**	**-**
***Citrobacter rodentium DBS 100***	**-**	**++**	**++**	**-**	**-**	**++**

(-, no activity; +, inhibition zone > 5-10 mm; ++, inhibition zone > 11-15 mm; +++, inhibition zone > 16-20 mm; ++++, inhibition zone > 20mm; DMSO, Negative control).

Measurement of inhibition zone was performed using disc diffusion assay. Each well was treated with a negative control and 20mg/ml concentrations of coumarin derivatives. *C. albicans, Candida albicans*; *B. cereus, Bacillus cereus*; *M. luteus, Micrococcus luteus*; *L. monocytogenes, Listeria monocytogenes*; *E. faecium, Enterococcus faecium*; *S. mutans, Streptococcus mutans*; *S. enteritidis, Salmonella enteritidis*; *S. boydii, Shigella boydii*; *E. coli, Escherichia coli*; *S. aures subsp. aureus, Staphylococcus aureus subsp. aureus*.

### 3.3 Selected coumarin derivatives induced cell membrane damage in bacteria

**Figure 1:**
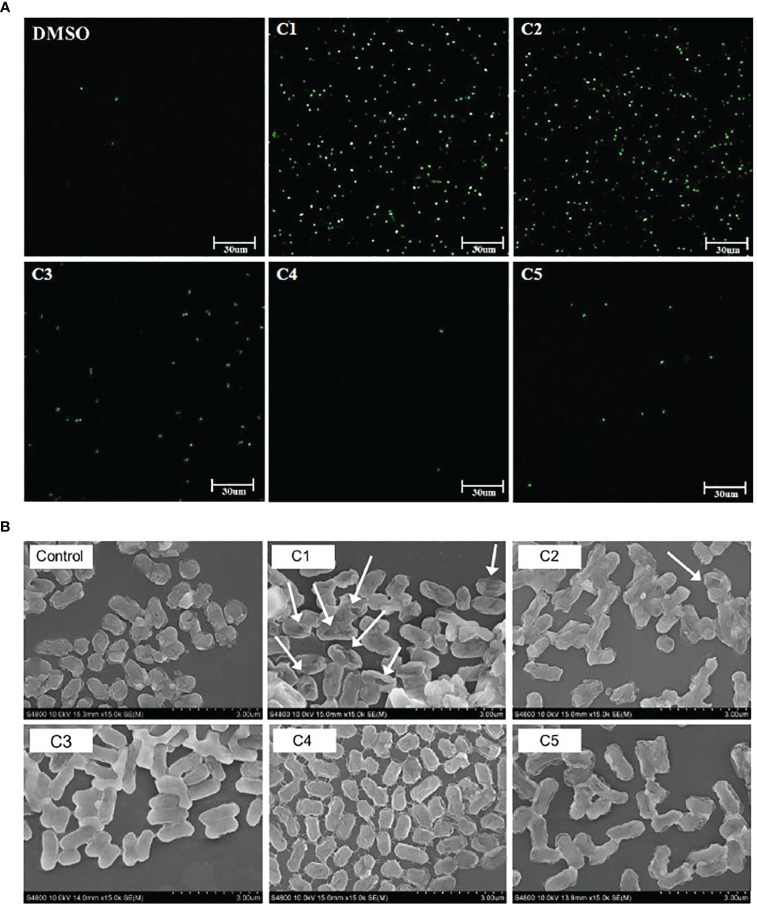
Effect of coumarin derivatives on membrane permeability of *C. rodentium*. **(A)** Effect of coumarin derivates on the integrity of the cell membrane of C. rodentium was determined via a Sytox Green assay. Scale bar = 30um. **(B)** Morphological characteristics of the membrane of *C. rodentium* was confirmed by SEM.

To explore the potential mechanisms of action of the coumarin derivatives with antibacterial activity, a SYTOX Green assay was performed. We evaluated the membrane permeability of *C. rodentium* following treatment with different coumarin derivatives. SYTOX Green cannot enter cells with intact cell membranes; however, if the cell membrane is damaged, it can pass through and bind to nucleic acids, increasing fluorescence (Lebaron et al., 1998). As shown in **Figure 1A**, fluorescence significantly increased in the C1 and C2 coumarin derivative-treated groups compared with that of DMSO group. However, C5 coumarin derivatives did not produce as many signals as C1 and C2 coumarin derivatives. Moreover, morphology of the *C. rodentium* obtained after exposure to the coumarin derivatives revealed significant modifications. It was confirmed that treatment with the C1 coumarin derivative had the greatest effect on the membrane of bacterial cells (**Figure 1B**). These results indicate that C1 and C2 coumarin derivatives killed the pathogen by causing obvious damage to the bacterial membrane, which could be a potential mechanism of action of the selected coumarin derivatives.

### 3.4 Protective effect of coumarin derivatives administration on the intestine after pathogen infection

**Figure 2:**
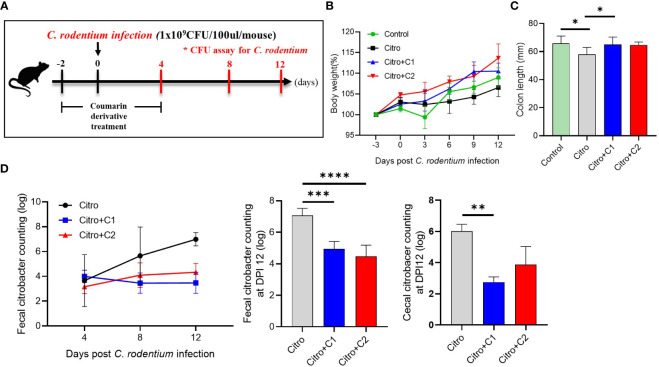
Protective effects of selected coumarin derivatives on *C. rodentium-*infected mice. C57BL/6 mice were randomly divided into four groups to examine the intestinal effects of selected coumarin derivates with excellent antibacterial activity. **(A)** Coumarin derivatives corresponding to each group were administered to mice in the coumarin derivatives-treated group once a day for 7 days beginning 2 days before *C. rodentium* infection. And mice in the control group and Citro group were treated with the same volume of distilled water. **(B)** Body weight changes throughout the entire duration of the study were measured, and **(C)** the colonic length was measured. **(D)** The fecal and cecal bacterial pathogen output was counted. Significance was determined by one-way ANOVA test (Tukey’s multiple comparison test). The data shown are the mean ± the SD (n = 4-5 mice/group/exp). At least 3 independent experiments were performed. Data from 2-3 independent experiments were combined. *, p<0.05, **, p<0.01, ***, p<0.001, ****, p<0.0001. Citro, *Citrobacter rodentium*.

To determine the further functions of coumarin derivatives C1 and C2, we examined body weight, colon length and pathogen numbers in the *C. rodentium* infectious-colitis model. Coumarin derivatives were administered to the treatment group for seven days, and all mice except the control group were subjected to *C. rodentium* infection (**Figure 2A**). Compared to the control group, the Citro group showed a relatively low increase in BW and the coumarin derivatives treated group showed a relatively large increase in BW, but there was no significant difference (**Figure 2B**). However, we confirmed that the colon length which had been reduced by infection, was restored by C1 coumarin derivatives treatment (**Figure 2C**). And coumarin derivatives reduced the pathogen (*C. rodentium*) load in the gut after infection. The number of pathogens in the feces of the Citro group gradually increased during the experimental period, whereas the coumarin-treated groups had a similar number of pathogens in the gut. At 12 days post-infection (DPI), C1 and C2 coumarin derivative-treated animals had significantly lower fecal pathogen numbers than the Citro group animals. The C1 coumarin derivative also reduced cecal pathogen numbers compared with the Citro group at 12 DPI (**Figure 2D**). These findings demonstrated that the administration of coumarin derivatives limited the expansion of *C. rodentium* in the gut.

### 3.5 Changes in the intestinal immune response in coumarin derivative-fed mice after pathogen infection

**Figure 3:**
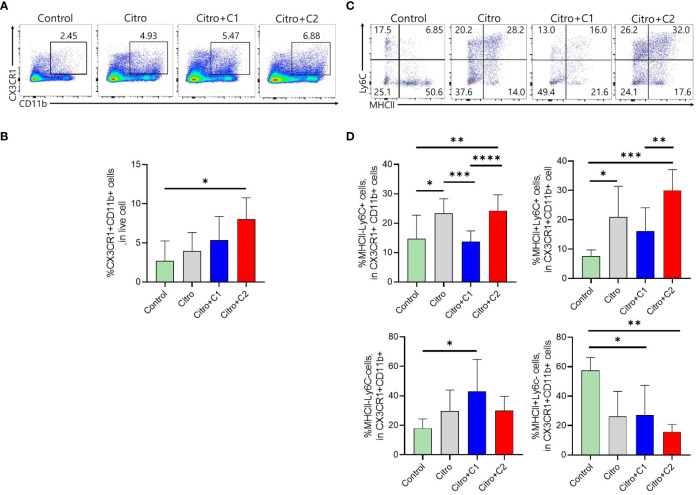
The effects of coumarin derivates on subsets of CX3CR1+CD11b+ MNPs cells population in the large intestine. **(A)** Representative flow cytometric analysis of colonic LP. Numbers in the contour area represent the percentage of cells in each gated area. **(B)** Statistical results of CX3CR1+CD11b+ cells in colonic LP in each group were shown. **(C)** Representative flow cytometric analysis gated with MHCII and Ly6C in the LP of each group was analyzed. **(D)** Flow cytometric analysis of CX3CD1+CD11b+ cells subsets gated with MHCll and Ly6C in the colonic LP of mice in each group was confirmed. Statistical results of MHCll-Ly6C-, MHCll-Ly6C+cells, MHCll+Ly6C+cells, MHCll+Ly6C-cells were analyzed in each group. Significance was determined by one-way ANOVA test (Tukey’s multiple comparison test). The data shown are the mean ± the SD (n=4-5 mice/group/exp). *, p<0.05, **, p<0.01, ***, p<0.001, ****, p<0.0001. At least 3 independent experiments were performed. Data from 2-3 independent experiments were combined.

**Figure 4:**
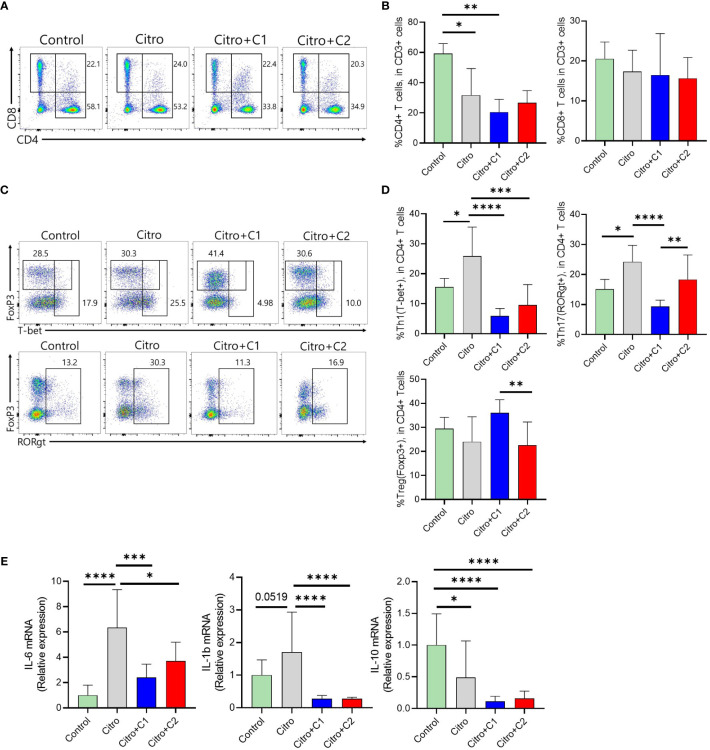
The effects of coumarin derivates on T cells and cytokine expressions in the large intestine with model of infectious colitis **(A)**. Representative flow cytometric analysis of CD4+ T cells and CD8+ T cells among CD3-gated T cells was confirmed in the intestinal tract of each group of mice. **(B)** Statistical analysis of CD4+ T cells and CD8+ T cells frequency was analyzed in total T cells. **(C)** Representative flow cytometric analysis of CD4+ T cell subsets was confirmed using the markers for each. **(D)** Statistical analysis of CD4+ T cells expressing T-bet, FoxP3, and RORrt among CD3-gated T cells was analyzed in the intestinal tract of each group of mice. **(E)** Colonic IL-6, IL-1b, IL-10 expression was determined by qRT-PCR. Significance was determined by one-way ANOVA test (Tukey’s multiple comparison test). The data shown are the mean ± SD (n=4-5 mice/group/exp). *, p<0.05, **, p<0.01, ***, p<0.001, ****, p<0.0001. At least 3 independent experiments were performed. Data from 2-3 independent experiments were combined.

The composition of immune cells was evaluated using flow cytometry to investigate the intestinal immune response after pathogen infection in the four groups. We examined CX3CR1+CD11b+ mononuclear phagocytes (MNPs) and CD4+ T cell populations in the LP of the intestine and MLN. First, we analyzed the proportion of CX3CR1+CD11b+ MNPs in the colonic LP (**Figure 3A**). The proportion of CX3CR1+CD11b+ cells in the LP was significantly increased in C2 coumarin derivative-fed animals compared with that in control animals. C1 treated animals did not differ in CX3CR1+CD11b+ cells than other groups (**Figure 3B**). We examined a subset of CX3CR1+CD11b+ cells expressing MHC II and Ly6C. The number of MHCll-Ly6C- cells was significantly increased in the Citro+C1 group. On the other side, the number of MHCll-Ly6C+ cells, which are potentially inflammatory monocytes, increased during infection compared to the control group and then significantly reduced in the Citro+C1 and Citro+C2 group. But, coumarin derivatives did not significantly alter the changes in MHCll+Ly6C+ and MHCll+Ly6C- cells (**Figures 3C, D**). Next, we checked whether the distribution of T cell subsets had changed. CD4+ cells were reduced by infection regardless of coumarin derivatives treatment and CD8+ T cells showed no significant differences among the four groups (**Figures 4A, B**). Significant differences in CD4+ T cell subsets were observed. The population of Th1 (CD4+T-bet+) cells, which increased due to infection, was significantly decreased by C1 and C2 administration. Also, the population of Th17 (CD4+RORgt+) cells, which increased by infection, showed a decreasing due to C1 treatment. However, the population of Tregs (CD4+Foxp3+) was not affected by the presence or absence of coumarin derivative treatment (**Figures 4C, D**). To assess the effect of coumarin derivatives on inflammation in intestinal tissue, the levels of inflammatory cytokines in colonic tissues were measured. The mRNA expression of IL-6 in colon tissue was significantly increased due to infection and the mRNA of IL-1b also tended to increase. And these inflammatory cytokine expressions were significantly decreased by C1 and C2 coumarin derivative treatment. However, coumarin derivatives did not affect IL-10 mRNA expression (**Figure 4E**). The same immune cells were examined in MLN. Unlike the colon results, there were no changes in the distribution of CX3CR1+CD11b+ MNPs and T cell subsets (**Supplementary Figures S1**, **S2**). Overall, the data indicated that compared to C2 coumarin derivatives, C1 coumarin derivatives effectively suppressed intestinal inflammation characterized by the suppression of monocyte expansion, inflammatory T cells, and inflammatory cytokine expression.

### 3.6 Administration of coumarin derivatives affects the gut microbiome shift by pathogen infection

**Figure 5:**
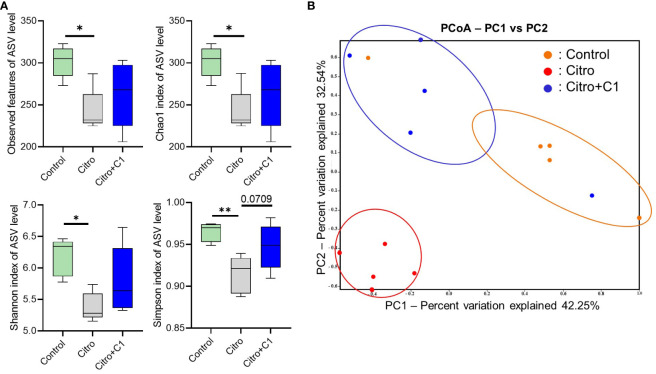
Diversity of the cecal microbiome in mice with or without coumarin derivatives treatment following *C. rodentium* infection. Cecal samples (n=5/group) were confirmed for microbial profile analysis using bacterial 16S rRNA gene sequencing. **(A)** Alpha diversity upon pathogen infection and C1 coumarin derivative treatment is displayed by the Observed feature, Chao1, Shannon, and Simpson index. **(B)** Beta diversity on microbial community composition between groups is shown by Principal co-ordinates analysis (PCoA). Significance was determined by one-way ANOVA test (Tukey’s multiple comparison test). The data shown are the mean ± SD (n=5 mice/group). *, p<0.05, **, p<0.001.

We investigated the effect of C1 coumarin derivatives, which showed the best regulatory effect on gut inflammation, on the cecal microbiota composition of *C. rodentium*-infected mice via 16S rRNA gene sequencing. Regarding alpha diversity, Observed features, Chao1, Shannon, and Simpson index of ASV levels were significantly reduced following pathogen infection. Although not significant, treatment with C1 coumarin derivative minimized the decline of the gut microbial diversity (**Figure 5A**). PCoA showed a separation in the gut microbiota structure among the three groups. Pathogen infection dramatically changed the microbiota compared with that in normal animals. And Coumarin-treated animals had a gut microbiome closer to that of control animals than *C. rodentium*-infected animals (**Figure 5B**).

**Figure 6:**
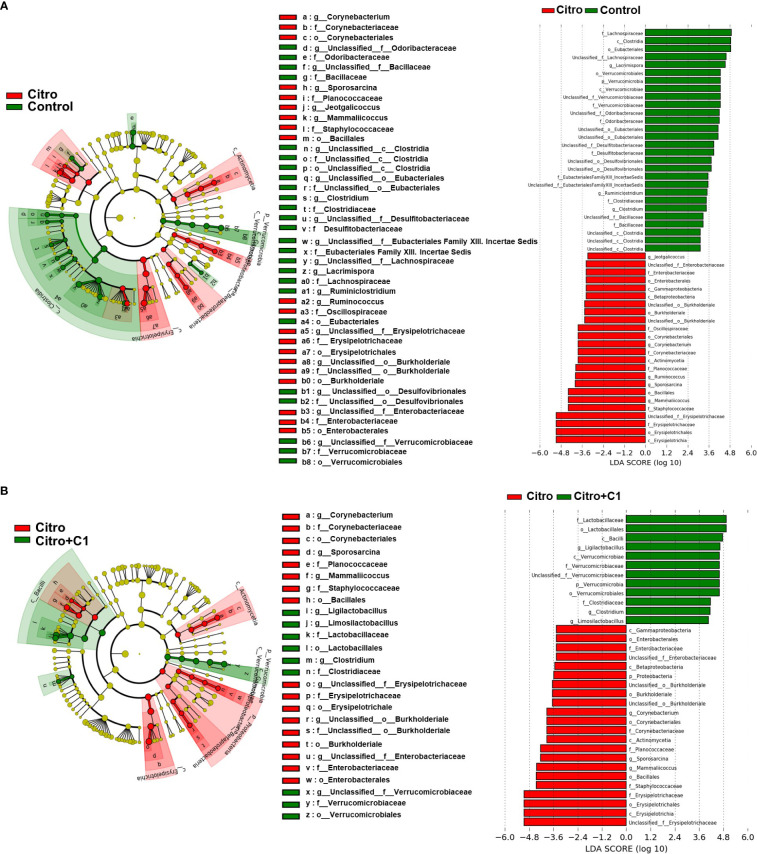
LEfSe analysis of microbial composition changes by pathogen infection and coumarin derivative treatment. Microbial differences in the taxa between **(A)** the non-infection (Control) group and the *C rodentium* infection (Citro) group, and **(B)** the *C rodentium* infection (Citro) group and *C. rodentium* infection with C1 coumarin derivative treatment (Citro+C1) group. Data were obtained from the control group (n=5), Citro group (n=5) and Citro+C1 group (n=5). (LDA > 2, *p* < 0.05).

Differentially abundant cecal bacterial taxa caused by pathogen infection and coumarin derivative treatment were identified using LEfSe analysis. We found that the abundance of pathogenic microbes such as *Enterobacteriaceae* and *Staphylococcaceae* was increased by *C. rodentium* infection (**Figure 6A**; **Supplementary Figure S3D**). Similarly, at the genus level, *C. rodentium*-infected mice displayed a significant increase in *Jeotgalicoccus* and *Mammaliicoccus* (**Figure 6A**; **Supplementary Figure S3E**). However, treatment with coumarin derivatives reduced the expansion of pathogenic microbes and promoted the proliferation of beneficial microbes. At the family level, *Verrucomicrobioaceae* were reduced by pathogen infection (**Figure 6A**) and recovered after treatment with coumarin derivatives (**Figure 6B**; **Supplementary Figure S3D**). At the genus level, three bacterial genera, *Limosilactobacillus*, *Ligilactobacillus*, and *Lactobacillus*, which belong to *Lactobacillaceae*, increased in the coumarin derivative-treated group (**Figure 6B**; **Supplementary Figure S3E**). These values were especially higher than those before pathogen infection (**Supplementary Figure S3E**). These results indicate that the C1 coumarin derivative attenuated *C. rodentium*-induced intestinal dysbiosis.

### 3.7 Effect of coumarin derivative supplementation on functional genes of the gut microbiome during pathogen infection

**Figure 7:**
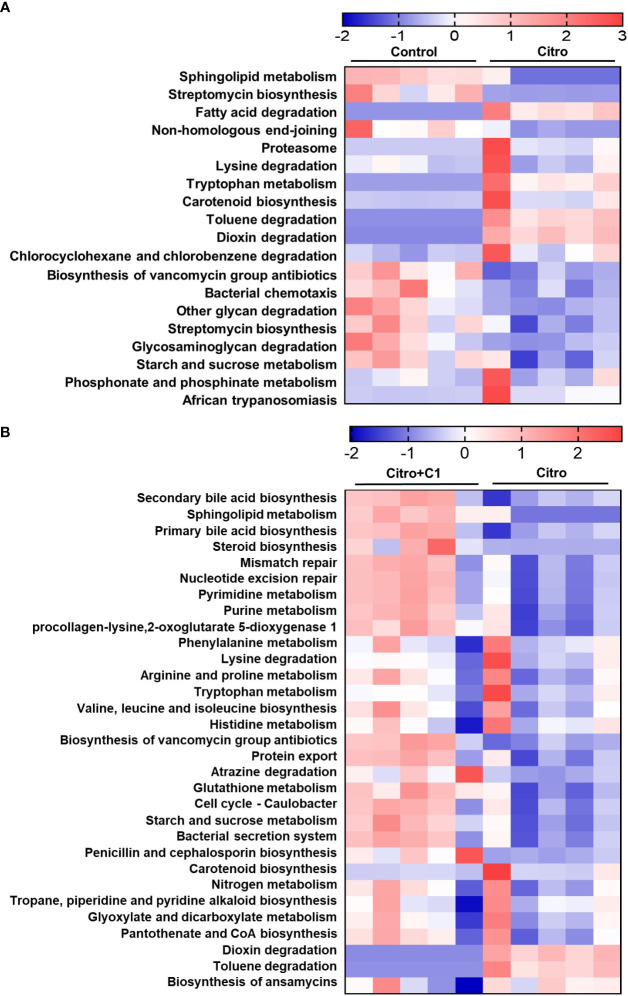
Changes of microbial functional gene enrichment by pathogen infection and treatment of coumarin derivative. The functional gene profiles of the microbial communities were predicted with the PICTUSt. Differences of microbial community functions between **(A)** the non-infection (Control) group and the *C. rodentium* infection (Citro) group, and **(B)** the *C. rodentium* infection (Citro) group and *C. rodentium* infection with C1 coumarin derivative treatment (Citro+C1) group. Data were obtained from the control group (n=5), Citro group (n=5) and Citro+C1 group (n=5).

We further analyzed changes in functional gene enrichment in the gut microbiome using PICRUSt 2 analysis. KEGG pathway analysis of the differentially expressed functions showed an abundance of signaling pathways (**Figure 7**). We first attempted to identify the altered functional genes following pathogen infection. Nineteen functional pathways that were altered by pathogen infection were identified. Lipid metabolism, including sphingolipid metabolism and steroid biosynthesis, was reduced by pathogen infection (**Figure 7A**). However, when infected mice were treated with coumarin derivatives, these metabolic pathways were restored, including sphingolipid, secondary bile acid, and primary bile acid biosynthesis (**Figure 7B**). There was no difference in the genes associated with nucleotide metabolism pathways after pathogen infection (**Figure 7A**), but metabolic pathways, such as pyrimidine and purine metabolism, increased after treatment with coumarin derivatives (**Figure 7B**). Pathogen infection downregulated the pathways associated with replication and repair, such as non-homologous end-joining (**Figure 7A**). However, functional pathways related to replication and repair, including mismatch repair and nucleotide excision repair, were upregulated following treatment with C1 derivative (**Figure 7B**).

To investigate the bacterial species responsible for the enrichment of functional genes triggered by dietary coumarin derivatives or pathogen infection-induced colitis, we used the FishTaco framework. This recently developed analytical method separates the predicted feature changes into taxon-level contributions with statistical significance (Douglas et al., 2020). An integrative analysis of taxon-level contributions to functional differences predicted an increase in the nucleotide excision repair pathway in the control group relative to the Citro group, and in the Citro+C1 group relative to the Citro group. *Limosilactobacillus* and *Lacrimispora* were the major drivers of nucleotide excision repair pathway enrichment under normal conditions. *Unc.Verrucomicrobia*, *Lactobacillus*, and *Ligilactobacillus* affected nucleotide excision repair pathway enrichment under Citro+C1 conditions (**Supplementary Figure S4B**). These results suggested that the functional shift in nucleotide excision repair due to changes in the intestinal environment is related to changes in the composition of the gut microbiome, such as *Unc.Verrucomicrobia*, *Lactobacillus*, and *Ligilactobacillus*. Overall, these results suggest that the mucosal microbiota of actively inflamed tissues may use different metabolic pathways than those of non-inflamed tissues.

## 4 Discussion

Environmental factors such as pathogen infection are closely related to intestinal health (Danese et al., 2004). Inflammation is the immediate biological response of the immune system to infection and irritation (Debnath et al., 2013). Pathogenic infections induce intestinal inflammation and disrupt intestinal tissue homeostasis (Meizlish et al., 2021). Hence, well-controlled luminal pathogens via gut immunity are essential for maintaining intestinal health. As the number of patients with pathogenic infections increases, the demand for treatment also increases (Baker et al., 2022). Especially because of the adverse side effects of first-line antibiotics, natural products have received attention as potential alternatives to these drugs (Atanasov et al., 2021). Compared with many studies on the anti-inflammatory properties of plant-derived extracts and derivatives (Guo et al., 2019), there is a shortage of studies supporting the efficacy of natural products in controlling gut inflammation, especially *in vivo* studies. Coumarin derivatives exhibit various biological properties. Research in the field of pharmacology has indicated that synthetic coumarin has an anti-inflammatory effect by inhibiting the production of pro-inflammatory cytokines in lipopolysaccharide-stimulated cells (Huang et al., 2012), antimicrobial effect by inhibiting bacterial metabolism and mechanisms of reproduction (Melliou et al., 2005), and antioxidant activity by inhibiting tissue damage (Kim et al., 2008). Here, we identified coumarin derivatives with antimicrobial activities and demonstrated their functional activities in suppressing pathogen-induced gut inflammation and pathogenic microbiome shifts by limiting pathogen expansion using an infectious colitis model.

We synthesized coumarin derivatives with various structures (**Table 1**) and found that the selected coumarin derivatives exhibited antimicrobial activity against various types of microbes. In the disc diffusion assay, the C1 coumarin derivatives showed the highest antimicrobial activity among all strains, followed by the C2 coumarin derivatives (**Table 2**). The C1 coumarin derivative possesses the structural feature of a trifluoromethyl group that contributes to its numerous biologically important molecular properties. Previous studies have shown that a coumarin derivative with a fluorine-containing CF_3_ group has very high antibacterial activity (Kalkhambkar et al., 2008). Synthesis of coumarin derivatives containing fluorine and a CF_3_ group is important for the development of natural antibacterial agents. As bacterial cell membranes separate and protect cells from the extracellular environment (Muheim et al., 2017), disruption of bacterial cell membrane integrity may affect the average growth and metabolism of bacteria (Wu et al., 2022), triggering bacterial death (Miki and Hardt, 2013). Therefore, various antibiotics have focused on bacterial membrane permeability (Ghai and Ghai, 2018). Several studies have shown that phenolic compounds (Farhadi et al., 2019) and coumarin derivatives (Huang et al., 2023) can target the bacterial cell membrane and combine with its phospholipid components. In this study, we used the SYTOX Green assay to determine whether coumarin derivatives exhibit pathogen-killing activity by destroying bacterial membranes. Additionally, SEM analysis was performed to confirm morphological images showing the destruction of the *C. rodentium* membrane. We demonstrated that C1 and C2 coumarin derivatives induced damage to the cell membrane in *C. rodentium* by assessing membrane integrity (**Figure 1**). Collectively, the cytomorphological observations showed that C1 and C2 coumarin derivatives have excellent antimicrobial activity via the disruption of bacterial cell membranes based on their pathogen killing activity.

To determine whether C1 and C2 coumarin derivatives can play a protective role against pathogen entry through their functional activities, an acute infectious colitis model was adopted. There was no significant difference in body weights (**Figure 2B**), but the colon length decreased by pathogen infection was recovered by C1 coumarin derivatives treatment (**Figure 2C**). There were no differences in the number of *C. rodentium* between the Citro group and coumarin derivative-treated groups until day 4. However, the number of *C. rodentium* cells in the feces of coumarin derivative-treated mice was lower after 8 days (DPI 8) and showed the greatest difference at DPI 12 in the feces from the colon and cecum (**Figure 2D**). This suggests that coumarin derivatives suppress the expansion of pathogens in the intestine after infection. Collectively, we demonstrated that coumarin derivatives exhibit antibacterial activity both *in vitro* and *in vivo*.

Pathogen expansion induces intestinal inflammation that damages the tissues (Perez-Lopez et al., 2016). Therefore, intestinal inflammation must be tightly controlled to prevent unnecessary tissue damage. Several studies have reported that plant extracts confer colonization resistance to intestinal pathogens through their anti-inflammatory effects (Nostro et al., 2006) (Naz et al., 2007). Intestinal immunity was examined to confirm the anti-inflammatory effects of the selected coumarin derivatives on the intestine during pathogenic bacterial infections. We used flow cytometry to identify the population of immune cells isolated from the LP layer, where most gut immune cells exist. In a steady intestinal state, Ly6C^hi^ monocytes are continuously recruited from the bloodstream to the intestine in a CCR2-dependent manner. Upon arrival at the mucosal membrane, they undergo various developmental processes. They exist as monocytes (MHCII-Ly6C+) and acquire MHCII to become mature monocytes (MHCII+Ly6C+). Subsequently, they lose Ly6C and become macrophages (MHCII+Ly6C-), as they undergo final differentiation into the majority of the population. Mucosal inflammation caused by pathogenic infections affects this process. There is an increased recruitment of MHCII-Ly6C+ monocytes to the infection site and an increase in inflammatory mediators such as TNF-α, IL-12, IL-23, IL-1b and IL-6, as well as activation of other inflammatory cells, including Th1 or Th17 cells (Varol et al., 2010). We examined this type of immune cell changes in our model. In examining the population of CX3CR1+CD11b+ MNPs (**Figure 3**), MHCII-Ly6C+ cells increased in Citro group compared to the control group but were significantly reduced in the C1 coumarin derivative-treated group (**Figure 3D**). Inflammatory monocytes express high levels of Ly6C, and a high monocyte level is also a sign of inflammation, infection, acute stress, blood disorders, and other health issues (Yang et al., 2014). Especially, it is known that the proportion of Ly6C+ monocytes increases in the intestine where colitis occurs (Jones et al., 2018). Therefore, it can be concluded that fewer inflammatory responses of innate immunity occurred in C1-supplemented animals. Changes in innate immunity, such as monocyte response, can affect T cell immunity during pathogen infection (Iwasaki and Medzhitov, 2015). It is well documented that Th1 and Th17 cells are induced in mice infected with pathogens such as *C. rodentium* (Jain et al., 2016). We further found that the distribution of Th1 cells (CD4+T-bet+) was significantly reduced in the Citro+C1 and Citro+C2 group compared with that in the Citro group. In addition, a decrease in Th17 cells (CD4+RORγt+), which mediate host defense mechanisms against various infections (Van De Veerdonk et al., 2009), was observed in the Citro+C1 group compared to the Citro group (**Figure 4D**). Inflammatory cytokines play a role in initiating the inflammatory response, regulating host defense against pathogens, and mediating innate and adaptive immune responses (Kany et al., 2019). In this study, coumarin derivative supplementation in *C. rodentium*-infected mice downregulated the cytokine genes for IL-6 and IL-1b, which were increased by infection in colonic tissues (**Figure 4E**). This is consistent with the results of a previous study showing that coumarin derivatives reduced intestinal inflammatory clinical indicators, accompanied by downregulation of colonic IL-6 (Di Stasi, 2021). IL-6 is produced in response to infections and tissue injuries, and contributes to host defense by stimulating acute phase responses (Tanaka et al., 2014). In addition, IL-6 is a key factor in the development of Th17 cells (Li et al., 2014), which play important roles against bacterial infection by recruiting acute inflammatory cells into the sites of infection (Curtis and Way, 2009). Also, IL-1b, which is increased by bacterial infection, exerts its protective action by recruiting the neutrophils to the site of inflammation, induction of chemokines and the stimulation of the Th17 response (Sahoo et al., 2011). Overall, the administration of C1 coumarin derivative attenuated intestinal inflammation by reducing pathogens that invaded the intestine, especially by focusing on suppressing the recruitment of inflammatory monocytes, differentiation of Th1 and Th17 cells, and mRNA expression of IL-6 and IL-1b cytokines.

Intestinal microbiota is essential for maintaining immunological homeostasis in the GI tract (Round and Mazmanian, 2009). Various extrinsic factors such as pathogen infection and diet influence the gut microbiome (Bajinka et al., 2020). This study also demonstrated that pathogen infection significantly affects the gut microbiome. *C. rodentium* infection significantly reduced community richness, and PCoA analyses revealed that the gut microbiota structure was significantly affected by pathogen infection. Notably, treatment with C1 coumarin derivative minimized these changes. Coumarin derivative treatment restored the richness and diversity of the gut microbiome (**Figure 5**). As diverse microbial ecosystems play a crucial role in preventing and resolving infectious diseases (Ubeda et al., 2017), increased gut microbiome diversity in C1 coumarin derivative-fed mice may benefit from gut inflammation. Using Linear discriminant analysis effect size (LEfSe) analysis, we identified differentially abundant taxa among the three groups at the genus level. Exposure to *C. rodentium* infection increased the populations of potential pathogenic microbes, such as *Enterobacteriaceae* and *Staphylococcaceae* at the family level, and *Jeotalicoccus* and *Mammaliicoccus* that belong to *Staphylococcaceae* at the genus level, compared with the control group (**Figure 6A**). The abundance of *Enterobacteriaceae* family is a typical signature of gut microbiota dysbiosis (Shin et al., 2015) and can neutralize the effects of existing antibacterial agents (Spera et al., 2019). And it is reported that *Staphylococcaceae* has a significant positive correlation with the expression of intestinal pro-inflammatory cytokines such as IL-1b in a colitis model (Ma et al., 2021). However, we confirmed that pathogenic microbes such as *Staphylococcaceae* and *Mammaliicoccus* were reduced by treatment with C1 coumarin derivative (**Figure 6B**). Our results are consistent with a study on polyphenolic compounds that ameliorated gut inflammation by preventing inflammatory cytokines IL-6 and IL-1b and restraining the growth of *Staphylococcaceae* (Yang et al., 2022b). Previous studies demonstrated that coumarin derivatives can modulate the antibiotic resistance against *Staphylococcaceae* and *Escherichia coli* (Martin et al., 2023). In particular, coumarin derivatives are known to control antibiotic resistance as an efflux pump inhibitor in the membrane of *Staphylococcaceae* (de Araújo et al., 2016; Martin et al., 2023). It has also been reported that antibiotic resistance can rapidly occur through changes in the expression of an efflux pump (Gaurav et al., 2023), so coumarin derivatives can be considered as potential therapeutic agents to restore the activity of antibiotics that have already lost their activity against bacteria. While treatment with C1 coumarin derivative reduced pathogenic microbial expansion, it increased the relative abundance of *Verrucomicrobiaceae* family, which is positively correlated with gut health and negatively correlated with gut inflammation (Ye et al., 2018). The abundance of the genera *Ligilactobacillus* and *Limosilactobacillus*, belonging to *Lactobacillaceae*, which are beneficial bacteria, also increased (**Figure 6B**). Multiple *in vitro* and *in vivo* studies have shown that these microbes exert anti-inflammatory activities (Yao et al., 2021) by regulating immune cells, reducing the production of Th1 cell and Th17 cell-associated cytokines (Liu et al., 2022), and enhancing the abundance of gut microbiota (Yao et al., 2021). In addition, considering that *Ligilactobacillus* increased when treated with C1 coumarin derivative after pathogen infection compared with that in the non-infected group (**Supplementary Figure S4A**), *Ligilactobacillus* is expected to increase even when treated with C1 coumarin derivative alone, regardless of inflammation, and play an important role in recovery from infectious inflammation. Furthermore, *Ligilactobacillus* is expected to be closely related to the anti-inflammatory response of C1 coumarin derivatives; however, further investigation is required to verify this mechanism in detail. Overall, it can be summarized that C1 coumarin derivative minimizes the pathological gut microbiome shift during pathogen infection.

Changes in the gut microbial diversity and composition lead to changes in microbial-derived functional gene enrichment. The regulation of colitis by plant extracts is closely related to the metabolic pathways involving gut microbiome genes (Ji et al., 2020). Moreover, synthetic coumarin derivatives exhibiting antibacterial properties affect the metabolic mechanisms of bacteria (Melliou et al., 2005). Lipid metabolism, including sphingolipid metabolism and steroid biosynthesis, was reduced by the pathogen infection (**Figure 7A**). However, treatment with C1 coumarin derivative not only restored these pathways but also increased primary and secondary bile acid biosynthesis belonging to lipid metabolism (**Figure 7B**). Consumption of polyphenol compounds is strongly associated with lipid metabolism in the gut microbiota (Ma et al., 2020) of mice (Li et al., 2022) and humans (Park, 2015). Previous studies have shown that sphingolipid metabolism regulates inflammatory signaling pathways (Maceyka and Spiegel, 2014). Sphingolipids can also affect inflammatory diseases by altering intestinal microbes (Norris et al., 2016) and acting as anti- or pro-inflammatory mediators (Chiricozzi et al., 2018). However, disruption of sphingolipid metabolism by pathogens neutralizes the host defense system (Wang et al., 2021). Previous studies also have shown that supplementation with *Ligilactobacillus* significantly increases the relative abundance of bacteria related to primary and secondary bile acid biosynthesis, thereby reducing gut microbiota dysbiosis (Liang et al., 2021). And the metabolic pathway of primary bile acids was improved by treatment with the phenolic compounds used in clinical practice (Yang et al., 2022a). There were also differences in nucleotide metabolism. Coumarin derivative-treated animals showed increased gene abundance for microbial functions related to nucleotide metabolism, including pyrimidine and purine metabolism (**Figure 7B**). It is known that nucleotide metabolism tends to increase in healthy people compared with patients with intestinal disease (Davenport et al., 2014). There were also differences in the replication and repair pathways between groups. The C1 coumarin derivative increased nucleotide excision repair and mismatch repair (**Figure 7B**), which are associated with repairs of damaged DNA pathways (Zhang et al., 2009). Infections caused by pathogenic microbes result in varying levels of inflammation and subsequent DNA damage (Sahan et al., 2018). This has the potential to cause mutations and inflammation in the surrounding cells, with the accumulation of DNA damage, ultimately leading to the risk of developing cancer (Kay et al., 2019). Therefore, pathways associated with DNA repair are essential for reducing inflammation. In line with these findings, our data showed that various gut microbial pathways serve as a mechanism for the positive effects of coumarin derivatives. This emphasizes the need for additional mechanistic studies to provide insights into the mechanisms by which metabolic changes resulting from coumarin derivatives with anti-inflammatory properties may contribute to infectious diseases. Functional prediction of the microbial community using 16S rRNA sequencing data and FishTaco analysis linked the C1 coumarin derivative-related bacterial taxa to the potential functional capabilities of microorganisms. According to FishTaco results comparing normal and pathogen infection conditions, the nucleotide excision repair pathway was altered in the cecal microbiome, and various microorganisms have been implicated (**Supplementary Figure S5A**). According to the conditions of coumarin derivative treatment after pathogen infection, FishTaco predicted that an increase in the relative abundance of *Unc.Verrucomicrobiaceae*, *Lactobacillus*, *Unc.Lachnospcraceae*, and *Ligilactobacillus* contributed to the increased relative abundance of the nucleotide excision repair pathway (**Supplementary Figure S5B**). Therefore, the nucleotide excision repair pathway decreased because of the increase in *Unc.Verrucomicrobiaceae* and *Ligilactobacillus* following treatment with the C1 coumarin derivative, which led to an intestinal anti-inflammatory effect. In summary, coumarin derivatives modulate gut microbiome composition during pathogenic infections. And overall, C1 coumarin derivatives have anti-inflammatory function and diminish pathogenic gut microbiome shift through antibacterial activity depending on pathogen-killing.

## 5 Conclusion

This *in vitro* study demonstrated a structure-dependent effect of coumarin derivatives on antimicrobial activity. Some of them showed antibacterial effects in the intestine with a pathogen infection-induced colitis model. In addition, selected coumarin derivatives suppressed the expansion of inflammatory immune cells, inflammatory cytokines, and pathogenic microbes induced by pathogen infection. Hence, coumarin derivatives have anti-inflammatory effects on the gut and improvement effects in the composition of gut microbiome during pathogen infection through antibacterial activity, suggesting a potential treatment for infectious diseases.

## Data availability statement

The datasets presented in this study can be found in online repositories. The names of the repository/repositories and accession number(s) can be found below: https://www.ncbi.nlm.nih.gov/, PRJNA1127804.

## Ethics statement

The animal study was approved by Institutional Animal Care and Use Committee (IACUC) of Pusan National University (PNU-IACUC; approval no. PNU-2023-0388). The study was conducted in accordance with the local legislation and institutional requirements.

## Author contributions

HJ: Writing – original draft, Writing – review & editing, Conceptualization, Data curation, Formal Analysis, Funding acquisition, Investigation, Methodology, Project administration, Resources, Software, Supervision, Validation, Visualization. YP: Data curation, Formal Analysis, Investigation, Methodology, Writing – original draft. B-HG: Data curation, Formal Analysis, Investigation, Methodology, Writing – original draft, Writing – review & editing. GH: Software, Writing – review & editing. WJ: Methodology, Writing – review & editing. SH: Data curation, Formal Analysis, Funding acquisition, Investigation, Methodology, Resources, Supervision, Visualization, Writing – original draft. MK: Writing – original draft, Writing – review & editing, Conceptualization, Data curation, Formal Analysis, Funding acquisition, Investigation, Methodology, Project administration, Resources, Software, Supervision, Validation, Visualization.
