# Supplementary material for: Coumarin derivatives ameliorate the intestinal inflammation and pathogenic gut microbiome changes in the model of infectious colitis through antibacterial activity
Source: Front Cell Infect Microbiol. 2024 Jul 15;14:1362773. doi: 10.3389/fcimb.2024.1362773 (PMC11287663; doi:10.3389/fcimb.2024.1362773)
Supplement: Supplementary file 1 [file DataSheet_1.docx]

Supplementary Material

# Supplementary Data

Supplementary Material should be uploaded separately on submission. Please include any supplementary data, figures and/or tables.

Supplementary material is not typeset so please ensure that all information is clearly presented, the appropriate caption is included in the file and not in the manuscript, and that the style conforms to the rest of the article.

# Supplementary Figures and Tables

For more information on Supplementary Material and for details on the different file types accepted, please see [here](https://www.frontiersin.org/guidelines/author-guidelines#supplementary-material).

## Supplementary Figures


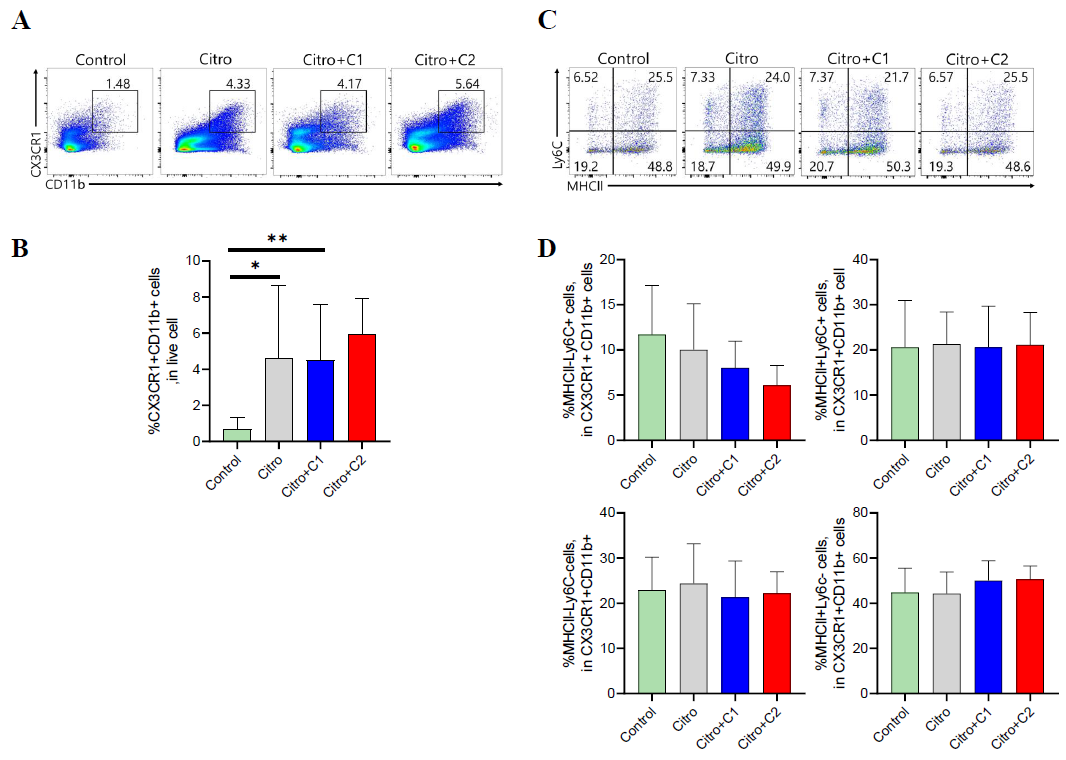


**Supplementary Figure 1.** **The effects of coumarin derivates on subsets of CX3CR1+CD11b+ mononuclear phagocytes (MNPs) cells population in the mesenteric lymph node (MLN)** (A) Representative flow cytometric analysis of MLN. Numbers in the contour area represent the percentage of cells in each gated area. (B) Statistics results of CX3CR1+CD11b+ cells in MLN in each group were analyzed. (C) Representative flow cytometric analysis of CX3CD1+CD11b+ cells subsets gated with MHCll and Ly6C in the MLN of mice was shown. (D) Statistics results of MHCll-Ly6C+cells, MHCll+Ly6C+ cells, MHCll+Ly6C-cells, MHCll-Ly6C-cells were analyzed. Significance was determined by one-way ANOVA test (Tukey’s multiple comparison test). The data shown are the mean ± the SD (n=4-5 mice/group/exp). At least 3 independent experiments were performed. Data from 2-3 independent experiments were combined.


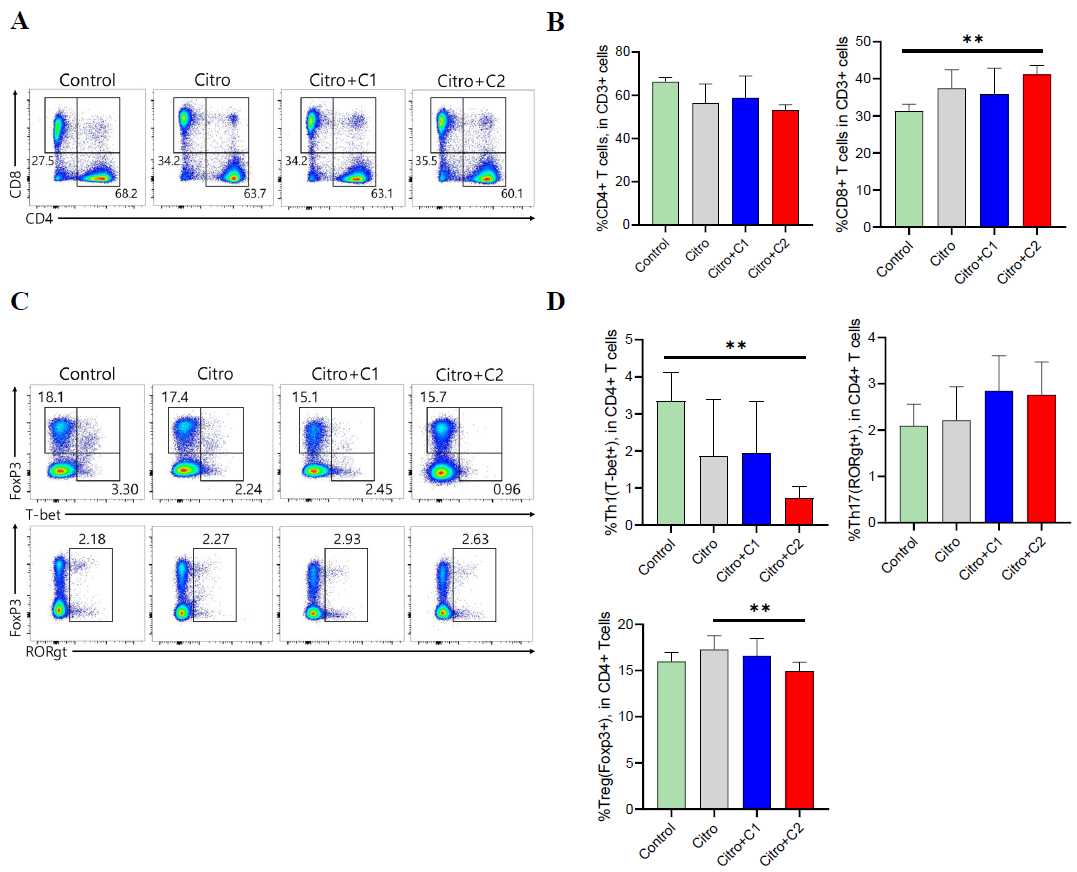


**Supplementary Figure 2. The effects of coumarin derivates on subsets of CD4+ T cells population in the mesenteric lymph node (MLN)** (A) Representative flow cytometric analysis of CD4+ T cells and CD8+ T cells among CD3-gated T cells in the MLN of each group was shown. (B) Statistical analysis of CD4+ T cells and CD8+ T cells frequency in total T cells was analyzed. (C) Representative flow cytometric analysis of CD4+ T cell subsets using the marker of each was confirmed. (D) Statistical analysis of CD4+ T cells expressing T-bet, FoxP3, and RORrt among CD3-gated T cells in the MLN of each group was analyzed. Significance was determined by one-way ANOVA test (Tukey’s multiple comparison test). The data shown are the mean ± SD (n=4-5 mice/group/exp). At least 3 independent experiments were performed. Data from 2-3 independent experiments were combined.


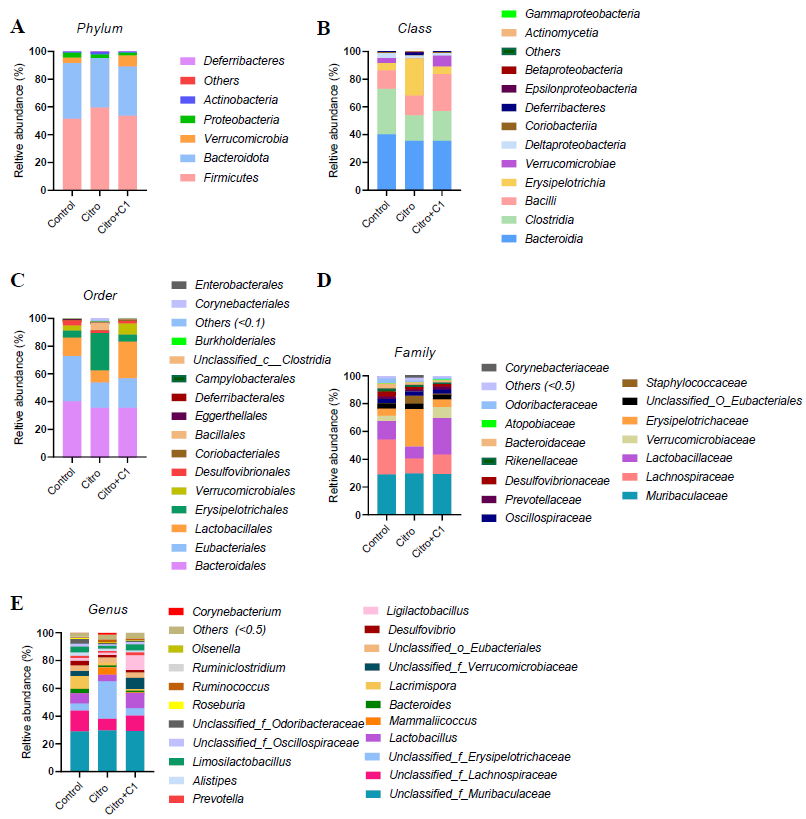


**Supplementary Figure 3. Coumarin derivative supplementation modulates gut microbiota composition during infection** 16S rRNA sequencing was conducted to determine the relative abundance at the phylum level (A), class level (B), order level (C), family level (D), and genus level (E). The proportion of the microbiome represented by less than 0.1% (phylum, class, and order level) and less than 0.5% (family and genus level) were classified as “Others.’’


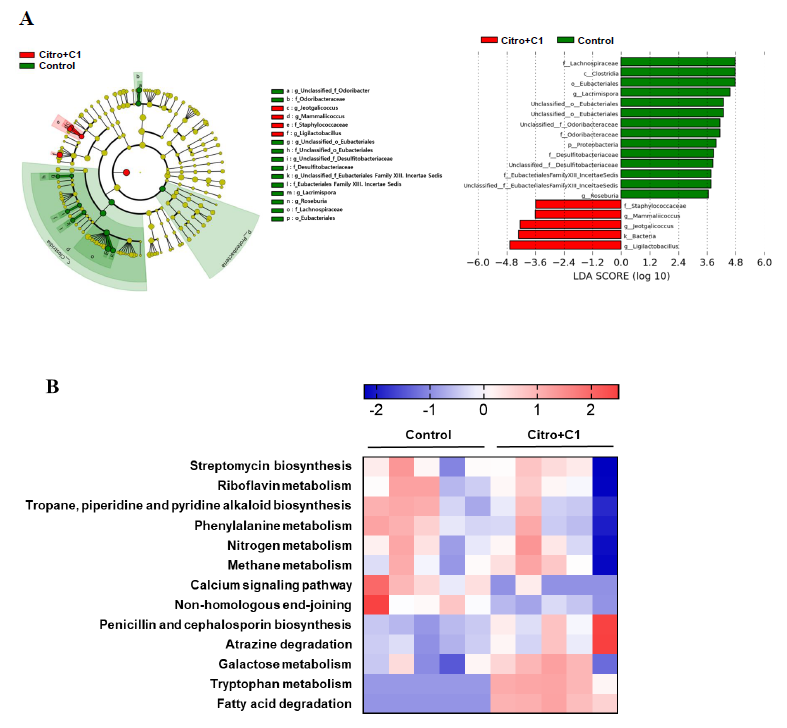


**Supplementary Figure 4. Changes of gut microbiome in mice treated with coumarin derivative after *C. rodentium*** (A) Linear discriminant analysis effect size (LEfSe) analysis on the cecal microbiome. Differentially abundant microbial distribution is shown for the control group and Citro+C1 group. A bar plot showing LDA scores of the genera that were differently abundant in the ceca microbiome. (B) Functional pathways enriched between the control group and the Citro+C1 group. Data were obtained from the control group (n=5) and Citro+C1 group (n=5). (LDA > 2, *p* < 0.05)


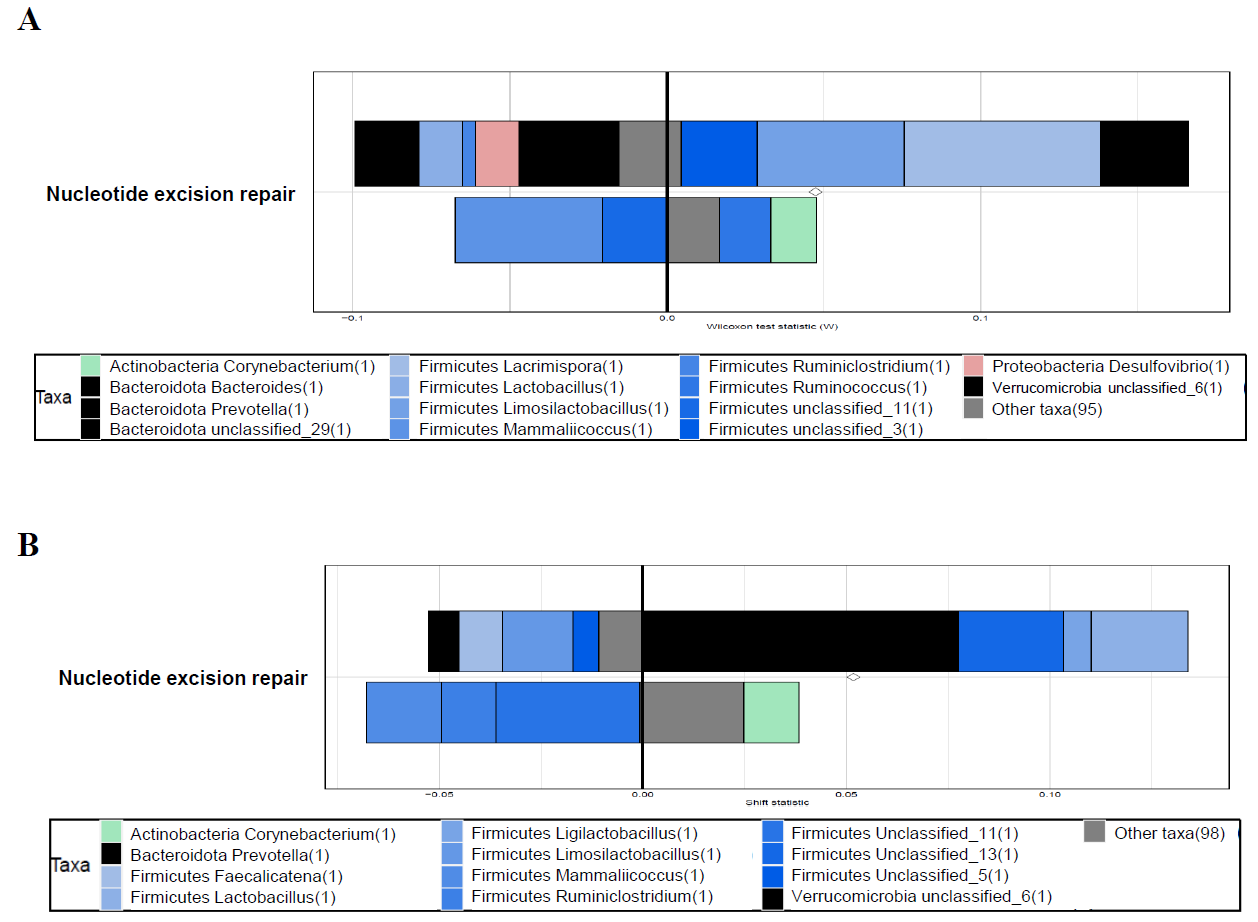


**Supplementary Figure 5. Microbiome-induced functional shift identified in cecal samples from mice by FishTaco** Taxonomic contributions to the shift in each function are shown as bars for functions enriched in the control group compared to the Citro group (A), and functions enriched in the Citro+C1 group compared to the Citro group (B). The length of the bar indicates the size of the contribution, and the position of the bar indicates the type of contribution. Depending on the microbial contribution, the top (bottom) bar in the enrichment group (control group for A, Citro+C1 group for B) indicates higher (lower) relative abundance. The contribution to the decrease (increase) in functional richness is indicated by the bars to the left (right) of the black vertical line.
